# Designing Studies for Epigenetic Biomarker Development in Autoimmune Rheumatic Diseases

**DOI:** 10.2478/rir-2022-0018

**Published:** 2022-10-20

**Authors:** Carlos de la Calle-Fabregat, Javier Rodríguez-Ubreva, Juan D. Cañete, Esteban Ballestar

**Affiliations:** 1 Epigenetics and Immune Disease Group, Josep Carreras Research Institute (IJC), 08916 Badalona, Barcelona, Spain; 2 Rheumatology Department, Arthritis Unit, Hospital Clinic and IDIBAPS, 08036 Barcelona, Spain; 3 Epigenetics in Inflammatory and Metabolic Diseases Laboratory, Health Science Center (HSC), East China Normal University (ECNU), Shanghai 200241, China

**Keywords:** epigenetics, biomarkers, inflammation, rheumatic diseases, autoimmune diseases

## Abstract

In just a few years, the number of epigenetic studies in autoimmune rheumatic and inflammatory diseases has greatly increased. This is in part due to the need of identifying additional determinants to genetics to explain the pathogenesis and development of these disorders. In this regard, epigenetics provides potential mechanisms that determine gene function, are linked to environmental factors, and could explain a wide range of phenotypic variability among patients with these diseases. Despite the high interest and number of studies describing epigenetic alterations under these conditions and exploring their relationship to various clinical aspects, few of the proposed biomarkers have yet reached clinical practice. The potential of epigenetic markers is high, as these alterations link measurable features with a number of biological traits. In the present article, we present published studies in the field, discuss some frequent limitations in the existing research, and propose a number of considerations that should be taken into account by those starting new projects in the field, with an aim to generate biomarkers that could make it into the clinics.

## Introduction

Epigenetics has become a recurrent term in translational and clinical research, which researchers and clinicians often use to provide additional or alternative (sometimes mysterious) mechanisms to genetics to explain the propensity to develop a disease. In the context of immune-mediated diseases, epigenetics is invoked in different contexts. For instance, in the context of monogenic immune conditions, also known as inborn errors of immunity (IEI), we can often find individuals with a pathogenic mutation with no symptoms, as well as individuals without mutations who do develop a clinical phenotype resembling patients with mutations.^[1]^ In this context, epigenetics could potentially explain both situations. In genetically complex immune-mediated diseases, such as most autoimmune rheumatic and inflammatory diseases, the presence of susceptibility variants does not always associate with disease, as it can be exemplified by genetically identical twins discordant for these conditions.^[2]^ Genome-wide association studies (GWAS) in autoimmune diseases have revealed single-nucleotide polymorphisms (SNPs) associated with susceptibility/protection to disease development, many of which are associated with gene regulatory genomic regions.^[3]^

A useful definition of epigenetic events describes them as the structural adaptation of chromosomal regions so as to register, signal, or perpetuate altered transcriptional activity states.^[4]^ The best studied epigenetic modifications are DNA methylation (DNAm) and histone post-translational modifications (PTMs).

DNAm is generally referred to as the addition of a methyl group to the 5th position of a cytosine, in the context of a cytosine followed by guanine (CpG) dinucleotide, leading to 5-methylcytosine (5mC). This modification is catalyzed by enzymes of the DNA methyltransferase (DNMT) family and is actively removed by a multi-step process that starts with oxidation of 5mC by ten-eleven translocation (TET) enzymes. DNMTs and TET have largely been shown to be indispensable for several biological processes such as development and cellular differentiation and activation, among others.^[5,6]^ Their importance in human biology is highlighted by the existence of mutations that are associated with a number of diseases due to an impairment of their enzymatic function,^[7,8,9]^ further underlining them as potential pharmacological targets in various pathogenic conditions.^[10]^

Histone PTMs result from the covalent modification of specific histone amino acid residues such as Lys, Arg, and Ser with different chemical groups, including acetyl, methyl, and phosphate, among others. Different histone modifications are introduced and removed by different families of histone acetyltransferases, deacetylases, methyltransferases, demethylases, kinases, phosphatases, etc.^[11]^

**Figure 1:**
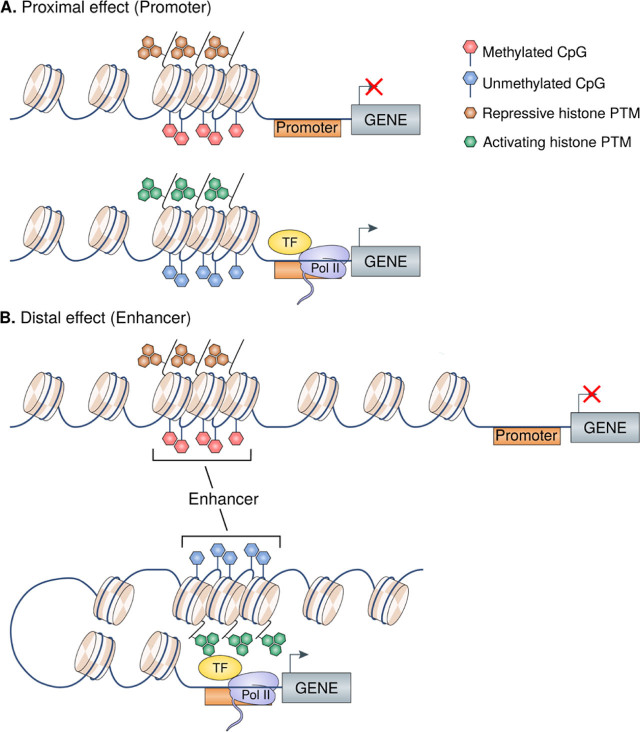
Conceptual models of epigenetic regulation of gene expression. A. Example of proximal promoter region regulated by both DNAm and histone PTMs. B. Example of distal enhancer region regulated by DNAm, histone PTMs, and physically interacting with the gene promoter of a gene. In both A and B, the top panel exemplifies a negative regulation (transcriptional silencing), while the bottom panel exemplifies a positive regulation (transcriptional activation). Pol II, RNA polymerase II; PTMs, post-translational modifications; TF, transcription factor.

Epigenetic marks determine or associate with the transcriptional status of genes and are targeted to specific genomic regions by different mechanisms, including the direct association of transcription factors (TFs) and upstream cell signaling pathways.^[6]^ TFs can directly recruit epigenetic enzymes and target epigenetic modifications to specific DNA sequences. Alternatively, TFs can indirectly influence or interfere with the recruitment of such enzymes to specific genomic regions. Some of these alterations influence or associate with gene expression changes in a proximal manner, such as those occurring in gene promoters, while others have long-distance effects, such as those taking place in enhancers (Figure 1). A critical characteristic of epigenetic modifications is their cell-specific nature, as they have a direct relationship with the particular transcriptional profile and function of each cell type.

**Figure 2:**
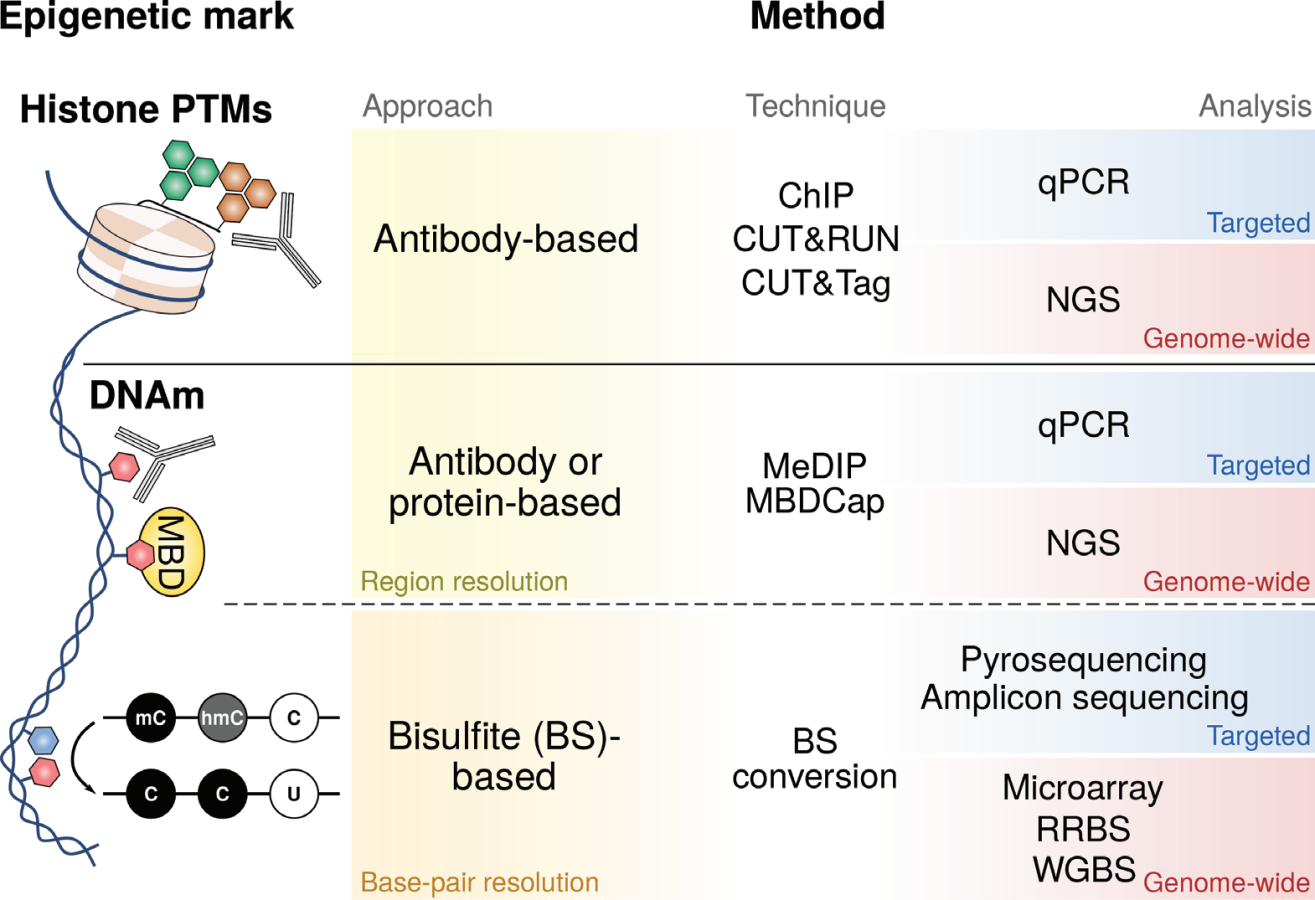
Summary of epigenetic profiling experimental techniques. Examples of most representative techniques for profiling histone PTMs and DNAm, highlighting the resolution of the technique (region or base-pair) and the extent of the analysis (targeted or genome wide). BS, sodium bisulfite; ChIP, chromatin immunoprecipitation; CUT&RUN, cleavage under targets and release using nuclease; CUT&Tag, cleavage under targets and tagmentation; DNAm, DNA methylation; hmC, hydroxymethylated cytosine; MBDCap, methyl-CpG binding domain (MBD)-based capture; mC, methylated cytosine; MeDIP, methylated DNA immunoprecipitation; PTMs, post-translational modifications; RRBS, reduced representation bisulfite sequencing; WGBS, whole-genome bisulfite sequencing.

There are a myriad of methods to analyze DNAm and histone modifications, either in a targeted manner or at a high-throughput or genomic level.^[12,13]^ Strategies include the targeted analysis of DNAm using bisulfite pyrosequencing or amplicon sequencing and histone modifications using chromatin immunoprecipitation (ChIP), cleavage under targets and release using nuclease (CUT&RUN) or tagmentation (CUT&Tag), coupled with quantitative PCR (qPCR) (Figure 2). For DNAm, high-throughput methods can be based on arrays, such as bead microarrays, or on the use of massive parallel DNA sequencing, such as whole genome bisulfite sequencing (WGBS) or reduced representation bisulfite sequencing (RRBS). All these techniques purvey distinct levels of genome coverage at a base-pair (single CpG) resolution, whereas other techniques based on sequence enrichment, such as methylated DNA immunoprecipitation (MeDIP) or methyl-CpG binding domain (MBD)-based capture (MBDCap), are limited to a region resolution, although usually at a lower cost^[14]^ (Figure 2). Histone modifications can be profiled genome wide by performing high-throughput sequencing of the material obtained from the previously mentioned techniques.

Given the relationship between epigenetic modifications, gene expression, and chromatin organization, the generation of maps of epigenetic modifications in both physiological and pathological situations, as well as their study in relation to the genomic sequence or transcriptional signatures, can help to understand the biological implications of such modifications.^[15]^ In addition, the analysis of these data in relation to different clinical aspects can be potentially relevant to obtaining biomarkers for different purposes.^[16,17]^

Among all epigenetic marks, DNAm is the most well studied. The analysis of DNAm is simpler than that of histone modifications because this chemical modification is directly located over the DNA sequence, which makes it highly stable in time and accessible in a wide range of isolated DNA material. Thus, DNAm data inherent robustness, added to its relatively easy interpretability, as well as to the aforementioned advantages with regards to its technical analysis, making it particularly convenient for large-scale clinical studies. In addition, DNAm datasets are the most numerous among all published epigenetic studies and have been used to recursively prove its suitability as biomarkers for several biological and clinical features.^[17,18]^

In this regard, most of the studies presented in this article focus on DNAm, although some selected articles that include histone modification data will also be mentioned. In recent years, many studies investigating the occurrence of epigenetic modifications in autoimmune and inflammatory diseases have been performed. A brief selection of studies is presented in the following section.

### Epigenetic Alterations in Autoimmune Rheumatic and Inflammatory Diseases: Clinical Implications

The analysis of epigenetic modifications in the field of autoimmune and inflammatory diseases has been performed for > 20 years.^[19]^ However, the majority of the studies have been published in the last decade, with increasing numbers of articles describing epigenetic signatures related to different clinical aspects such as disease subtype, prognosis, disease activity and response to treatment, among others. Most of these studies have been performed in genetically complex autoimmune diseases, although there are also examples in monogenic inflammatory syndromes.^[6]^ In this section, we will present a brief selection of highlights in the context of autoimmune rheumatic diseases, focusing on studies where epigenetic alterations are inspected in relation to clinical features, with a focus on DNAm.

Initially, many studies focused on the identification of existing epigenetic alterations in a given condition. For instance, high-throughput screenings have identified DNAm alterations in autoimmune rheumatic conditions such as systemic lupus erythematosus (SLE),^[20]^ rheumatoid arthritis (RA),^[21]^ systemic sclerosis (SSc),^[22]^ and primary Sjögren's syndrome (SjS).^[23]^ More recently, different studies have outlined alterations in specific immune cell types that were previously associated with these diseases, including B and T lymphocytes^[24]^ and monocytes^[25]^ as well as non-immune cell types also relevant to the disease pathophysiology (e.g., synovial fibroblasts [SFs] in RA). ^[26]^ DNAm alterations in specific cell types and subpopulations are associated with different pathogenic functions including hyperactivation in B and T cells, as well as the generation of inflammatory responses.

A major aim of modern medical practice is to use tools to classify patients in the most accurate and precise manner. Biomarkers can help to distinguish those disease subtypes and anticipate patient progression and response to treatments.^[27]^ Different studies have addressed the relationship between DNAm and disease subtype in various autoimmune rheumatic diseases, revealing differences between patients with renal affection^[28]^ or cutaneous manifestations^[29]^ in SLE, between anti-citrullinated peptide antibody (ACPA)-positive and APCA-negative patients in RA^[30]^ and between limited versus cutaneous SSc,^[31]^ among others. Interestingly, besides aiding in the classification of patients at the time of diagnosis, DNAm profiles have been proposed as a prognostic biomarker in early undifferentiated stages of arthritis (UA) to anticipate the progression into differentiated forms of the disease.^[32,33]^

Disease activity is another relevant feature in relation to clinical care. Autoimmune diseases are characterized by periods of high activity (or “flares”) that alternate with periods of remission, which is the status aimed by clinicians to become sustained under specific therapies. For some diseases, there are accurate activity indexes that allow a precise assessment and monitoring of the patient's status. However, autoimmune rheumatic diseases are usually characterized by a complex pathogenesis, which makes it difficult to find robust biomarkers to monitor disease activity. Different studies have found an association between DNAm signatures and disease activity in RA,^[25]^ UA,^[33]^ and SLE,^[34]^ suggesting the possibility of generating activity biomarkers for these diseases.

In addition, several pieces of evidence suggest that DNAm could also be used as a predictor of treatment response. These findings represent not only a benefit of patient well-being but also an improved usage of resources by the public health systems. Examples of this include the identification of markers for response to methotrexate, the first-line disease-modifying antirheumatic drug (DMARD) to control active inflammation in RA patients,^[35]^ or for the response to biological treatments, such as tumor necrosis factor (TNF) inhibitors, also in RA.^[36]^

The existence of significant associations between epigenetic profiles and different types of clinical features supports the usefulness of epigenetic marks to better define patient clinical status and provide standard methods, which could be applied in the clinics.

### Common Limitations of Epigenetic Studies as Clinical Biomarkers

Despite the increasing number of epigenetic studies in autoimmune and inflammatory diseases, a number of limitations have not facilitated the transfer of the obtained results to clinical practice. General limitations of the different studies have occurred at different levels. These include the extent of the analysis (targeted regions versus genome-wide coverage), the nature of the biological sample (whole tissue versus isolated cell populations), the amount of the collected material, and the size of the cohort.

In relation to the first point, initial epigenetic studies started before high-throughput methods for epigenomic screening were available and, therefore, focused on specific genes or regions. Some of these studies were of great value to explore the idea of epigenetic dysregulation in autoimmune disease. However, the possibility of screening hundreds or thousands of genes at a time was only possible with the advent of arrayand next-generation sequencing-based technologies.^[14]^

Many initial studies on DNAm were mainly performed using whole tissues, which in immunology research (almost entirely) corresponds to using whole blood cells (WBCs) or peripheral blood mononuclear cell (PBMC) samples. There are several obvious reasons to explain it. First, some of these studies made use of patients’ cohort samples originally collected for genetic studies or at least not considering the high cell-type specificity of epigenetic profiles and, therefore, its potential for identifying biologically meaningful alterations. On the other hand, for most clinical units, it is much more feasible to preserve whole blood or PBMC samples rather than to isolate specific cell types. In technically limited environments, generating epigenetic profiles from those data has been a way to identify general signatures that still might be of great clinical and epidemiological interest. Moreover, disease feature-associated cell-type-specific signatures can still be partially retrieved from those data with the use of deconvolution algorithms, which allow to estimate alterations undergoing in the different cell types that constitute a complex sample.^[37]^ However, these approaches consist of theoretical approximations, and thus, their conclusions can never be fully equated to those from pure cell-type data studies.

Another determining condition for performing epigenetic studies is the amount of available biological material, which might limit the performance of some of the available assays. Considering that, if the cell type subjected to study is rare or its numbers are reduced under pathological conditions, this could be an additional limitation. For instance, in a study performed by our team, we aimed to investigate the DNA methylomes of B cell subpopulations from patients with primary immunodeficiency. In this context, memory B cells are substantially reduced and, at the same time, constitute the cell subpopulation where most of the changes occur. This limitation was overcome by using a single-cell-based approach (single-cell bisulfite sequencing [scBS-seq]),^[38]^ which allowed us to generate extensive DNAm profiles of memory B cells, in a biologically scarce sample.^[39]^ Furthermore, these approaches are of utmost utility when applied on highly heterogeneous material, where it allows a characterization of the cellular complexity. However, single-cell methods for DNAm profiling are still costly and require considerable technical and computational infrastructure.^[40]^

The size of the cohorts has been a major limitation for most studies. In contrast to GWAS, which are generally performed with vast cohorts of patients, epigenome-wide association studies (EWAS) of comparable cohort sizes are scarce. In addition to that, epigenome datasets are influenced by a number of confounding factors that can impact epigenetic modifications including age, gender, pharmacological treatments, and lifestyle-associated parameters. In this regard, basic epidemiological considerations are sometimes overlooked during the experimental design. These should be addressed to ensure the credibility and reproducibility of the results before proceeding to sample analysis. The possibility of improving epigenetic studies in relation to autoimmune diseases, or to any monogenic or genetically complex diseases, would require the consideration of a well-designed cohort with a size that allows determining the effect of potential confounding factors, analyzing all potential pathogenic cell types, as well as understanding their mutual interactions. In addition, those epigenetic studies would benefit from the use of screening methods that allow understanding the main contributors and readers (e.g., TFs or signaling pathways) of the existing dysregulation in order to develop sensitive and biologically meaningful markers. Ideally, these studies should not only focus on epigenomics but also constitute multi-omic approaches including genomics, transcriptomics, proteomics, and metabolomics. Such a study would require enormous economic efforts and very well-coordinated force tasks. In the following section, we provide an itemized description of those considerations, which we hope helps prioritize different factors in relation to the needs and the capabilities of different laboratories.

### Designing and Planning Meaningful Studies

**Figure 3:**
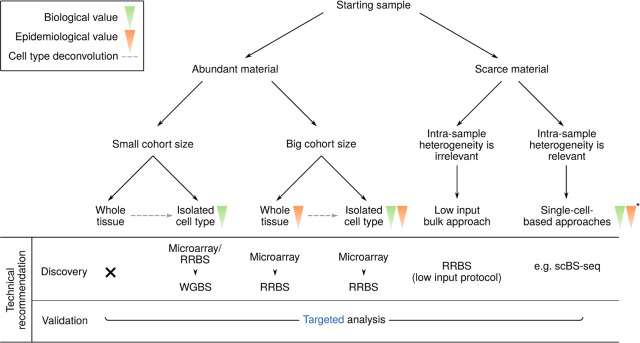
Technical recommendations before designing studies on DNAm. Decision tree diagram considering several points regarding experimental design such as (i) amount of material, (ii) cohort size, (iii) type of material, and (iv) intra-sample heterogeneity. A technical recommendation list is suggested in every case. *Single-cell-based approach scalability is still restricted due to elevated costs, which might limit epidemiological studies. DNAm, DNA methylation; RRBS, reduced representation bisulfite sequencing; scBS-seq, single-cell bisulfite sequencing; WGBS, whole genome bisulfite sequencing.

There are no infallible protocols for the design of studies for biomarker identification in autoimmune and inflammatory diseases, and the specific requirements will depend on the purpose of each study. However, there are several general and specific considerations that can be considered and various elements that should be addressed. These include decisions related to the design of the cohort in terms of size and composition (to minimize confounding factors for epigenetics analysis), sample preparation and storage, and the choice of epigenomic profiling method. Technical recommendations considering several points are summarized in Figure 3.

In relation to the design of the cohort, regardless of the type of study to perform (disease classification, prognosis, activity, or drug response), a first obvious recommendation is to balance the study groups in your cohort by all available epidemiological variables (e.g., age, sex, smoking status, and others) as well as in relation to other potential confounding factors.^[41]^ For instance, DNAm profiles are highly affected by age. In fact, there are multiple reports describing the acquisition of methylation changes with age.^[42,43]^ This also holds true for sex, ethnical background,^[44]^ and cigarette smoke,^[45]^ among others. All these parameters can be used as a covariate during the differential methylation analysis to control for potential confounding effects and improve the statistical power.^[46,47]^ However, to be able to do so, it is pivotal to work with a cohort that includes a wide range of distribution of all these variables and that these are overlapping and ideally balanced among all comparison groups.

Also, pharmacological treatments are relevant for epigenomic studies. Drugs such as DMARDs, corticosteroids, and other molecules used as co-adjuvants (such as vitamin D) in autoimmune and inflammatory conditions are known to affect DNAm profiles.^[48,49,50]^ This is of particular interest for drug response studies, where ideally patients should be recruited at baseline before entering the treatment for testing. In some cases, it is unfeasible to select patients that are fully treatment naïve since sometimes patients arrive at the specialist after visits with general practitioners, who provide first-line treatment. For all these reasons, patient treatment profile and history needs to be considered during the design of the cohort and during the analysis of the data.

Besides all the aforementioned characteristics, other aspects in the design of the cohort that are relevant to specific studies include additional clinical parameters such as the disease activity, the presence of autoantibodies, or the patient comorbidities, among others. Researchers should make sure to collect the maximum available clinically relevant information before proceeding to analysis. In this sense, studies with large sample sizes and a deep characterization of the patients will allow not only to obtain more robust results but also to control better for all these additional covariates as well as to find specific associations with all of them, individually.

In line with this, the size of the cohort is highly determinant to identify significant and reproducible differences. Minimal cohort size requirements can be estimated prior to analysis by performing a power analysis, which will provide sample amount cut-offs to reach statistical significance. Of note, there are existing power analysis tools that are specifically tailored for DNAm profiling through Illumina microarrays.^[51,52]^

In genetic studies, the source of biological material can be secondary, except in cases where specific populations are searched for somatic mutations or mosaicisms. In contrast, in epigenetic studies, the tissue or cell type and decisions related to sample preparation and storage are highly relevant. First, it is important to know the cell type subjected to epigenomic analyses. In some cases, it is possible to obtain biopsies of the affected material, such as synovial tissue or fluid from RA patients or salivary glands from SjS patients. However, in other cases, only blood might be available, and then, it is essential to decide whether the epigenetic status of blood cell types is relevant or not for the study. In the case of inflammatory conditions, in some cases, inflammation is systemic and can then be inspected directly from blood, although if inflammation only occurs locally, its impact in peripheral blood cells might be irrelevant.

In case blood samples are collected, it would be relevant to isolate one or several immune cell types to ensure the specificity of the analysis. One or several cell types should be selected depending on their biological or pathological relevance to the condition. For instance, monocytes and monocyte-derived cells are highly sensitive to inflammatory environmental influences.^[6]^ The selection of the cell type to analyze can be based on existing studies, either from flow cytometry and/or single-cell sequencing-based datasets, that help identify the specific cell subpopulation to study.

Sample storage should be considered with prospective means. By default, it is recommended to cryopreserve the material, particularly if the future method of analysis is not fully defined at the time of sample collection. This process, however, can lead to increased cell mortality,^[53]^ and ideally, processing fresh material is the best option if the protocol is defined beforehand. If the analysis of choice is DNAm, directly freezing the sample (either in bulk or after cell isolation) should be enough. Double extraction of DNA and RNA should also be considered. If epigenetic marks to be analyzed are histone modifications, samples should be cross-linked before storage to ensure that the interaction between histone modifications and the DNA is preserved in the chromatin.

The material amount will also be relevant for downstream experiments. The selection of the method of analysis in relation to the amount of input material can be determined in pilot tests. This is particularly relevant for rare cell populations, in which single-cell-based methods might be the best option. However, if the intra-sample cellular heterogeneity is not of interest for the study, bulk methods with low input protocols (e.g., RRBS low-input protocol)^[54]^ might be better alternatives. If the available material is too low to be applicable for any high-throughput technique, it can always be considered to be used for validation of previous results.

Different methods can be used to perform DNAm profiling, including bead arrays, RRBS, or WGBS. For large cohorts, it is always advisable to use either EPIC arrays, which are easy to use and economic, or targeted amplicon sequencing, based on prior data generated with high-throughput screening methods. In the case of histone modifications, ChIP-seq, CUT&RUN, or CUT&Tag methods can be used, and the methods need to be decided before starting the collection of samples to establish the method of sample storage.

In summary, there are a number of aspects that should be considered before starting a project aiming at generating epigenomic datasets for biomarker development that can be tailored to the specific aims of the study.

### Perspectives for Future Studies

The future of the use of biomarkers based on epigenetic modifications highly relies on the development of studies that consider both general aspects relative to the proper design of the cohort type and size and particular requirements of epigenetic studies which need to be cell-type specific and may require special conditions for cell isolation and preservation.

Although there are available tools to optimize the analysis of datasets, it is of inherent interest to urge the scientific and clinical community to spend efforts on well-designed studies that consider both the general and specific needs of these studies. Given the size of the cohorts, this will likely require the coordinated efforts of both scientific and clinical teams.
